# The Effects of Intranasal Oxytocin on Neural and Behavioral Responses to Social Touch in the Form of Massage

**DOI:** 10.3389/fnins.2020.589878

**Published:** 2020-12-04

**Authors:** Yuanshu Chen, Qin Li, Qianqian Zhang, Juan Kou, Yingying Zhang, Han Cui, Jennifer Wernicke, Christian Montag, Benjamin Becker, Keith M. Kendrick, Shuxia Yao

**Affiliations:** ^1^The Clinical Hospital of Chengdu Brain Science Institute, MOE Key Laboratory for NeuroInformation, Center for Information in Medicine, University of Electronic Science and Technology of China, Chengdu, China; ^2^Department of Molecular Psychology, Institute of Psychology and Education, Ulm University, Ulm, Germany

**Keywords:** oxytocin, social massage, reward response, autism traits, fMRI

## Abstract

Manually-administered massage can potently increase endogenous oxytocin concentrations and neural activity in social cognition and reward regions and intranasal oxytocin can increase the pleasantness of social touch. In the present study, we investigated whether intranasal oxytocin modulates behavioral and neural responses to foot massage applied manually or by machine using a randomized placebo-controlled within-subject pharmaco-fMRI design. 46 male participants underwent blocks of massage of each type where they both received and imagined receiving the massage. Intranasal oxytocin significantly increased subjective pleasantness ratings of the manual but not the machine massage and neural responses in key regions involved in reward (orbitofrontal cortex, dorsal striatum and ventral tegmental area), social cognition (superior temporal sulcus and inferior parietal lobule), emotion and salience (amygdala and anterior cingulate and insula) and default mode networks (medial prefrontal cortex, parahippocampal gyrus, posterior cingulate, and precuneus) as well as a number of sensory and motor processing regions. Both neural and behavioral effects of oxytocin occurred independent of whether subjects thought the massage was applied by a male or female masseur. These findings support the importance of oxytocin for enhancing positive behavioral and neural responses to social touch in the form of manually administered massage and that a combination of intranasal oxytocin and massage may have therapeutic potential in autism.

**Clinical Trials Registration:**

The Effects of Oxytocin on Social Touch; registration ID: NCT03278860; URL: https://clinicaltrials.gov/ct2/show/NCT03278860.

## Introduction

Although language-based communication plays a dominant role in human social interaction, interpersonal social touch is also important for helping to create and maintain social bonds through affective communication (Dunbar, 2010; Cascio et al., 2019). The sense of touch relies on the sensory and perceptual consequences of stimulation of low-threshold afferent fibers in the skin since these cutaneous afferents convey distinct sensory/perceptual qualities which are projected to different stimulus-specific neurons (McGlone et al., 2014). There are multiple types of nervous fibers that transmit cutaneous stimulation, including both fast conducting thick myelinated A-β afferents which subserve the discriminative perception of touch (i.e., the pressure, vibration, and texture) and thin unmyelinated low-conducting nerve receptors including C- and A-δ afferent fibers which are described as thermo-, noci- or chemoreceptive (Björnsdotter et al., 2010; McGlone et al., 2012, 2014; Takahashi et al., 2015). The low-threshold unmyelinated C-touch afferent fibers (or CT fibers), which selectively respond to gentle, slowly applied caress-like stroking on hairy skin (Ackerley et al., 2014; McGlone et al., 2014), are found to associate closely with pleasantness processing of social affective touch (Essick et al., 2010; Pawling et al., 2017) and increased activity in corresponding brain reward regions such as the orbitofrontal cortex (OFC) and other regions involved in social-affective processing such as the insula and the superior temporal sulcus (STS) (Olausson et al., 2010; Gordon et al., 2013; Björnsdotter et al., 2014; Croy et al., 2016; Morrison, 2016).

Tactile stimulation, particularly those via CT fiber afferents, have been proposed as cutaneous mediators for release of the hypothalamic neuropeptide oxytocin (OT) during social tactile interactions (Uvnäs-Moberg et al., 2015; Walker et al., 2017) and OT plays a key role in modulating social interactions and affective processing across species (Bartz et al., 2011; Kendrick et al., 2017). Tactile stimulation, including stroking, social grooming and social affective touch, have been found to facilitate OT release and thus increase the OT concentrations in blood, saliva, or urine across species (Mitsui et al., 2011; Schneiderman et al., 2012; Crockford et al., 2013; Uvnäs-Moberg et al., 2015; Vittner et al., 2018). More specifically, plasma OT levels in rats were increased dramatically when their backs or abdomen were gently stroked (Stock and Uvnäs-Moberg, 1988; Uvnäs-Moberg and Petersson, 2010). In humans, blood OT concentrations in post-partum mothers increase to some extent in response to skin-to-skin contact which involves several kinds of touch/stroking (also massage like hand movement on the mothers’ breast) during mother-infant interactions when a radioimmunoassay (RIA) is used (Matthiesen et al., 2001; Uvnäs-Moberg et al., 2015, 2020). Uvnäs-Moberg et al. (2020) also propose that skin-to-skin contact stimulates the activity in the oxytocinergic system and thereby reduces stress and promotes functions linked to restoration and growth of social interactive relationships. “Warm touch” between couples and affectionate touch during early stage of romantic relationships at a certain frequency can also increase plasma OT concentrations (see Light et al., 2005; Holt-Lunstad et al., 2008; Schneiderman et al., 2012). Furthermore, a number of studies have reported decreased basal OT concentrations in autism spectrum disorder, which is characterized by problems in social interaction and communication as well as altered tolerance of social touch (Kanner, 1943), and basal OT concentrations are negatively correlated with autistic symptom severity in both clinical and healthy populations (Parker et al., 2014; Taurines et al., 2014; Zhang et al., 2016).

Massage applied with a moderate pressure is also a pleasant form of affective touch and has great potential in therapeutic applications including reducing pain and stress and enhancing immune function, which may additionally activate non-C-type fibers as it has been shown to be related to the stimulation of pressure receptors under the skin (Field, 2010, 2019). Animal models have provided evidence that massage-like stroking to rats could produce an analgesic effect, with reduced blood pressure and cortisol levels (Agren et al., 1995). A study by Lund et al. (2002) which demonstrated this analgesic effect of massage-like treatments, also reported that it significantly increased plasma and periaqueductal OT levels. Furthermore, human studies have also shown that slow, moderate pressure massage can facilitate endogenous OT release (Morhenn et al., 2012; Li et al., 2019). More specifically, there is direct evidence showing that manually applied, moderate pressure massage of the top of the foot can increase plasma OT concentrations in men and that only massage by hand, as opposed to by machine, increases activity in key brain regions involved in processing hedonic values (OFC) and social cognition (STS) aspects of affective touch (Li et al., 2019). Preliminary clinical evidence for massage-evoked increases in OT concentrations has also been reported in autistic children (Tsuji et al., 2015). Thus social touch, especially administered as massage, can be effective as a non-invasive strategy for enhancing endogenous OT release and therefore of potential therapeutic benefit in autism and other psychiatric disorders exhibiting social dysfunction and reduced basal OT levels.

While previous studies have demonstrated a close association between OT release and massage, none to date have directly examined the modulatory effects of exogenous OT administration on behavioral and neural responses to it. Scheele et al. (2014a) have reported that intranasal administration of OT increased perceived pleasantness and activation of the OFC and insula in response to positive social touch applied to the leg in Caucasian male subjects. This facilitatory effect of OT on social touch was highly context-dependent, however, and only occurred when the male subjects thought the touch was administered by a female and not by a male (Scheele et al., 2014a). A subsequent study in the context of romantic relationships found similar effects of OT only when subjects thought the touch was applied by their partner and not by an unfamiliar person of the opposite sex (Kreuder et al., 2017). By further excluding social intention and relationship context, a more recent study using well-controlled CT-targeted affective touch with different valences has revealed a general effect of intranasal OT on increasing both the perceived pleasantness of touch and OFC activation independent of touch valence (Chen et al., 2020). Thus, we hypothesized that intranasal administration of OT would enhance the perceived pleasantness of massage, and associated brain reward responses, independent of who administers the massage, since subjects are mainly responding to the hedonic properties of being touched manually independent of the identity of the person administering it.

The current study therefore investigated whether intranasal OT would modulate behavioral and neural responses to CT-targeted massage of the foot using a within subject pharmaco-functional MRI combined approach in healthy subjects. Social (manual) compared with machine administered massage were used to test whether effects of OT were social-context dependent. Based on the bio-informational theory proposed by Lang (1977, 1979), mental imagery can evoke similar responses to real interaction with a stimulus and can be used as a therapeutic strategy. Subsequent studies have revealed similar emotional and neural responses in imagined and real interactions with emotional stimuli (Kim et al., 2007; Costa et al., 2010; Lewis et al., 2013; Ji et al., 2016). In addition to core regions of the touch processing network overlap with the “mirror neuron” system (notably the insula and STS) which responds equivalently to real and imagined stimuli (Rizzolatti and Fabbri-Destro, 2008), and a recent study in humans has reported that parietal cortex neurons can respond equivalently to both real and imagined touch (Chivukula et al., 2020). We therefore additionally included imagined massage in the two conditions (imagined manual versus imagined machine massage). We anticipated that this would provide proof-of-concept evidence for the feasibility of using imagined massage as a self-administered substitute for real massage in possible future therapeutic applications. Additionally, blood samples were taken to measure basal plasma OT concentrations and autistic traits were measured using the Autism Spectrum Quotient (ASQ – Baron-Cohen et al., 2001) to investigate their respective associations with effects of intranasal OT on individual responses to massage. Based on the demonstrated facilitatory effects of OT on social and affective touch (Scheele et al., 2014a; Chen et al., 2020) and previous findings that manual massage increased OT release (Morhenn et al., 2012; Li et al., 2019), we hypothesized that intranasal OT would significantly increase the perceived pleasantness and corresponding activity in brain regions associated with social touch processing in response to real or imagined massage administered manually but not by machine. Given the negative correlations between OT-induced behavioral and neural effects to touch and autistic traits and the negative correlations between massage-evoked OT concentration changes and autistic traits (Scheele et al., 2014a; Li et al., 2019), we additionally hypothesized that OT-induced responses to massage would be associated with basal OT concentrations in the blood and trait autism.

## Materials and Methods

### Participants and Procedures

50 healthy male subjects (mean age = 21.22 years old, *sd* = 2.77) participated in the present study. Exclusion criteria consisted of any psychiatric/physical disorders, alcohol/substance abuse, or other major health concern. Written informed consent was obtained from all subjects before the experiment. All procedures in the study were approved by the local ethics committee at the University of Electronic Science and Technology of China and were in line with the latest revision of the declaration of Helsinki. Four subjects were excluded due to excessive head movement (>3 mm) in either one or both of the scanning sessions. Thus, data from a final total of 46 subjects were analyzed.

### Blood Samples and OT Assay

Blood sample collection and OT assay methods were as previously described (Li et al., 2019). Briefly, before the treatment, blood samples (6 ml) were collected from all subjects by venipuncture for measurement of basal OT concentrations in plasma using a commercial ELISA (Cayman Chemical, Ann Arbor, MI, United States Kit 500440). Samples were analyzed in triplicate and a standard prior extraction step was performed in accordance with the manufacturers recommended protocol and spiked samples (with 100 pg/ml OT added) were included with every assay to calculate extraction efficiency which was 96%. The extraction step incorporated a twofold concentration of samples using a vacuum concentrator (Concentrator plus, Eppendorf, Germany) resulting in a detection sensitivity of 3 pg/ml. All samples had detectable concentrations. The intra- and inter-assay coefficients of variation were 6 and 8%, respectively. The manufacturer’s reported cross-reactivity of the antibody with related neuropeptides, such as vasopressin and vasotocin, is <0.01%.

### Questionnaire Assessments of Behavioral Traits and Mood

Subjects were asked to complete Chinese versions of validated questionnaires on personality, traits, attitude toward interpersonal touch and sensitivity to reward. Personality trait measures included the Beck Depression Inventory II (BDI-II) (Beck et al., 1996), the State-Trait Anxiety Inventory (STAI) (Spielberger et al., 1983), the Autism Spectrum Quotient (ASQ) (Baron-Cohen et al., 2001), the Liebowitz Social Anxiety Scale (LSAS) (Heimberg et al., 1999) and the Empathy Quotient (EQ) (Baron-Cohen and Wheelwright, 2004). The Social Touch Questionnaire (STQ) (Wilhelm et al., 2001) and the Sensitivity to Punishment and Sensitivity to Reward Questionnaire (SPSRQ) (Torrubia et al., 2001) were used to assess individual sensitivity to touch and reward and sensory responsivity was assessed by the Sensory Over-Responsivity (SensOR) Scales (Schoen et al., 2008). To control for potentially confounding effects of OT on mood, all subjects completed the Positive and Negative Affect Schedule (PANAS) (Watson et al., 1988) immediately before and 35 min after the treatment. Additionally, the PANAS was administered after each type of massage to measure the influence of massage on mood. After completing all the questionnaires, participants were asked to wash their feet before the massage experiment in the scanner. Following a randomized, double-blind, placebo-controlled within-subject design, subjects underwent two fMRI assessments (with an interval >2 weeks) and were randomly assigned to receive either the intranasal OT (24 IU; Oxytocin Spray, Sichuan Meike Pharmaceutical Co. Ltd., China) or placebo (PLC, identical ingredients except the peptide – i.e., glycerine and sodium chloride) administration. Treatment-order was counterbalanced and subjects were unable to guess better than chance whether they had received OT or PLC (χ^2^ = 0.77, *P* = 0.38).

### Experimental Design

The massage task started 45 min after the nasal spray administration in line with evidence for the time course of OT effects (Paloyelis et al., 2016; Spengler et al., 2017). A professional masseur blinded to the research aim was trained by the experimenter to keep the velocity and pressure of massage as constant as possible during the experiment. The task was composed of 4 massage conditions: social (manual), imagined social, machine (administered via boots) and imagined machine massage. Participants were informed that a male masseur or a female masseuse would be randomly assigned in the MRI room to deliver the foot massage in the manual massage condition (in reality the massage was always given by the same male masseur), and that the machine massage was applied by a commercial foot massage machine which involved them wearing boots on each foot. All subjects reported that they had no preference for the gender of the masseur during the telephone interview before being recruited in the study. Since the electric foot massage boot machines were not magnetic resonance imaging compatible, the masseur was trained to apply massage to them in a mechanical way to imitate the machine-based massage and to apply it with approximately the same force and velocity as the manual massage. In post-scan interviews, subjects all believed that the machine massage was indeed administered by a machine and not by a person. In the imagined massage conditions, subjects were asked to try their best to imagine they were receiving manually delivered massage by the masseur (imagined manual massage) or machine massage (imagined machine massage).

**FIGURE 1:**
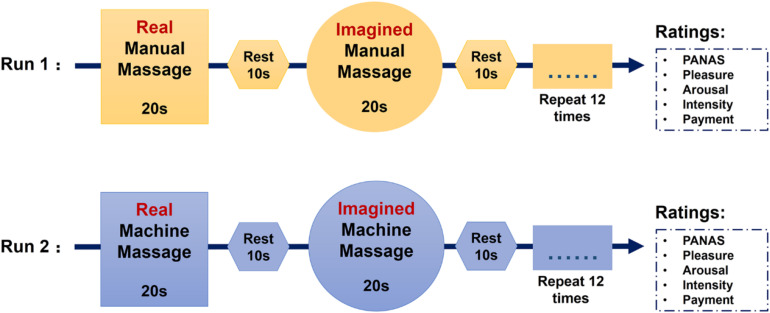
Sequence of the massage task.

There were two functional runs in the massage task (12.5 min for each run). In the manual massage run, there were 12 blocks of manual massage and 12 blocks of imagined manual massage with each block lasting for 20 s. Each manual massage block was followed by an imagined manual massage block alternating with a rest period of 10 s. In the machine massage run, there were also 12 machine massage blocks and 12 imagined machine massage blocks with each block lasting for 20 s. Each machine massage block was followed by an imagined machine massage block alternating with a 10 s rest (Figure 1). We chose a design where subjects were required to imagine being massaged immediately after they had received a real massage in order to make it easier for them to be able to imagine the experience accurately. The order of manual and machine massage runs was counterbalanced across subjects. Massage was delivered on both feet simultaneously to control for possible preferences for left or right and more importantly to induce bilateral neural responses. Note that the massage used in the present study is relatively different from the clinical massage which is usually applied in a more continuous way and is sometimes combined with the use of massage oil (see Espí-López et al., 2016; Nasiri et al., 2016; Perlman et al., 2019). The current experimental design of massage application was primarily intended to demonstrate detailed effects of OT on massage-evoked changes in brain and behavior before establishing a potentially clinically applicable protocol. The use of short periods of massage alternating with periods of rest allowed us to conduct an event-related fMRI analysis where we could have repeated observations of the neural effects of massage during each run. This repetition thereby increases confidence in the overall findings compared with a single long period of continuous massage. Although foot massage may be not used often in western cultures, this type of massage regimen is very common and popular in China and other Asian cultures. We also chose to use foot massage in the current study since it is more feasible to apply massage simultaneously to both feet while subjects lay within the MRI scanner and because of our previous findings that foot massage can increase oxytocin concentrations in blood as well as neural responses in the OFC and STS using a similar paradigm to the present study (Li et al., 2019).

During the scanning, cues indicating massage conditions were presented on the screen to inform to the participant which type of massage they would receive. Subjects were instructed to lie in the scanner quietly and focus on the applied massage. Immediately after each run, subjects completed the PANAS again and then answered the following four questions about the massage: (1) How pleasant did you feel the massage was? (1 = extremely unpleasant, 9 = extremely pleasant). (2) How much did the massage arouse you? (1 = drowsy and unresponsive, 9 = very aroused). (3) How intense was the massage? (1 = extremely light, 9 = extremely strong) and (4) How much would you be willing to pay if you had to pay for the massage? Please choose from 1 to 100 (1 = 1 RMB, 9 = 100 RMB). Massage periods and types were indicated to the masseur simultaneously by a second monitor. The entire opening of the scanner was covered with a blanket so that subjects could not see their feet or the masseur. After scanning, participants were asked to guess whether the manual massage was applied by a male or female professional masseur to control for possible sex-dependent effects.

### fMRI Image Acquisition and Data Analyses

A 3.0 Tesla GE Discovery MR750 system (General Electric Medical System, Milwaukee, WI, United States) was used to obtain T2^∗^-weighted echo-planar pulse sequence (TR = 2,000 ms, TE = 30 ms, slices: 43, slice thickness: 3.2 mm, field of view: 220 mm × 220 mm, matrix size: 64 × 64, flip angle: 90°). In addition, high revolution T1-weighted anatomical images were acquired obliquely with a 3D spoiled gradient echo pulse sequence (TR = 6 ms, TE = minimum, slices: 156, slice thickness: 1 mm, field of view: 256 mm × 256 mm, matrix size: 256 × 256, flip angle: 12°). Images were processed using SPM12 software (Wellcome Department of Cognitive Neurology, London^1^) (Friston et al., 1994). The first five functional volumes were discarded to achieve magnet-steady images. Images were corrected for head movement using the 6-parameter rigid body algorithm. After co-registering the mean functional image and the T1 image, the T1 image was segmented to determine the parameters for normalizing the functional images to the Montreal Neurological Institute (MNI) space. The normalized images were finally spatially smoothed using a Gaussian kernel (8 mm full-width at half maximum).

The first level design matrix included 6 regressors (real manual massage, imagined manual massage, real machine massage, imagined machine massage, the rest before the real massage and before the imagined massage) and the six head-motion parameters convolved with the canonical hemodynamic response function as confounding variables. Contrast images for each condition minus the rest and combined social/machine massage (real and imagined) minus the rest were created for the OT and PLC sessions separately. Only the rest period before the real massage was used as the control condition to avoid possible confounding effects of carry-over from the real massage. On the second level, one sample *T*-tests on corresponding contrasts were used to test massage-specific effects and paired *T*-tests were used to compare treatment differences. A significance threshold of *P* < 0.025 false discovery rate (FDR) corrected at peak level was set for multiple comparisons for whole brain level analyses. Parameter estimates were extracted for each subject from a 6-mm sphere centered at the maximum *t*-value of treatment differences to plot and calculate associations between OT-induced neural effects and basal OT concentrations and trait autism using the Pearson correlation.

## Results

### Questionnaires

**FIGURE 2:**
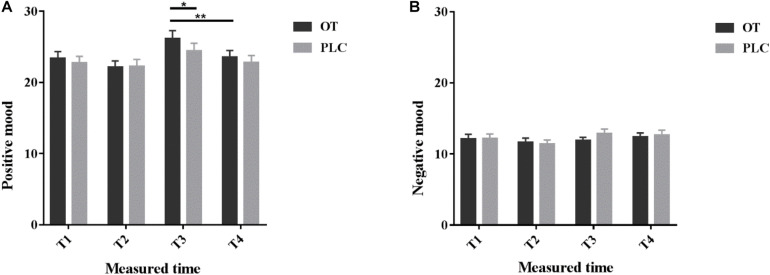
Positive and Negative Affect Schedule scores. Individual **(A)** positive and **(B)** negative mood scores before (T1), 35 min after (T2) intranasal treatment and also immediately after the manual massage (T3) as well as after the machine massage (T4) with oxytocin (OT) or placebo (PLC) in the MRI scanner. The order of the massage runs was counterbalanced. *p < 0.05, **p < 0.01.

Questionnaire scores are reported in Supplementary Table 1. All subjects’ scores on anxiety, depression and social anxiety were within the normal range. To explore whether intranasal OT treatment and massage had effects on personal mood, we analyzed individual self-reported mood scores measured by the PANAS. Comparison of pre- and post-treatment PANAS scores revealed no significant influence of treatment *per se* on either positive (*p* = 0.408) or negative mood scores (*p* = 0.09). However, OT treatment significantly increased positive mood ratings after receiving the manual massage relative to the machine massage (*p* = 0.006), but not after PLC treatment (*p* = 0.07, see Figure 2). After the manual massage when subjects had received OT treatment they had significantly higher positive mood scores compared after PLC treatment (*p* = 0.015, see Figure 2) (details see Supplementary Materials). This may indicate that under OT subjects generally experienced a more positive mood following the manual but not the machine massage.

### Behavioral and Neural Responses to Massage Under PLC

In order to investigate the general effect of massage on behavioral and neural response to real and imagined massage given manually or by machine, we first analyzed the data from the 46 subjects under PLC treatment before subsequently investigating the effects of OT. Under PLC, subjects rated manual as opposed to machine massage as more pleasant (mean ± SEM of rating scores: manual = 6.00 ± 0.19 vs. machine = 5.28 ± 0.23; *t*_45_ = −2.726, *p* = 0.009), arousing (manual = 5.65 ± 0.21 vs. machine = 4.35 ± 0.20; *t*_45_ = −5.132, *p* < 0.001) and intense (pressure) (manual = 5.63 ± 0.19 vs. machine = 4.48 ± 0.16; *t*_45_ = −4.709, *p* < 0.001). Subjects were also willing to pay more for the manual than the machine massage (manual = 5.57 ± 0.28 vs. machine = 3.67 ± 0.25; *t*_45_ = −6.203; *p* < 0.001). We also explored whether the perceived gender of the masseur would influence ratings of the massage. Based on the post-scanning interview, a total of 24 subjects reported that they thought they had received the massage from a male and 22 from a female, demonstrating clearly that they were unable to guess the gender accurately (χ^2^ = 0.09, *p* = 0.77). Pleasantness ratings did not differ significantly between subjects who thought the manual massage was given by a male or by a female (*t*_44_ = −1.416, *p* = 0.164).

At the neural level, a whole brain level analysis revealed that the only significant differences in responses to real as opposed to imagined manual massage were mainly in motor control regions (paracentral lobe, pre- and post-central gyrus, cerebellum and supplementary motor area) but not in the social touch processing networks. A similar pattern was seen between difference of the real and imagined machine massage (see Table 1). Thus imagined manual massage may induce similar neural responses to the real manual massage. To increase statistical power, we therefore combined the real and imagined massage conditions. A whole brain analysis on the combined real and imagined manual massage under PLC also revealed increased activation in the somatosensory and motor regions (cerebellum, paracentral lobule, pre-central gyrus, post-central gyrus and supplementary motor area, supramarginal gyrus) as well as the in social cognition (the STS, inferior parietal lobule) and visual areas (lingual gyrus) (see Table 2). For the combined real and imagined machine massage, there was also increased activation in the somatosensory and motor regions (paracentral lobe, post-central gyrus, cerebellum) as well as in the posterior default mode network (precuneus) and visual processing regions (fusiform gyrus) (see Table 2). Comparisons at the whole brain level between combined real and imagined massage given manually or by machine revealed that manual massage produced significantly greater activation in brain reward (the OFC), salience (anterior cingulate cortex-ACC and anterior insula-AI) and social cognition (STS) regions, as well as in the medial and lateral prefrontal cortex (mPFC and lPFC), default mode and attentional networks and motor regions (cerebellum) (see Table 2).

**TABLE 1:** Whole brain neural responses to real and imagined massage under PLC.

Brain region	Voxels	Peak-*t* value	*x*	*y*	*z*
**Real social > imagined social**					
R. postcentral gyrus	816	13.02	12	−42	75
L. postcentral gyrus		10.95	−15	−42	75
R. precentral		8.45	9	−21	75
R. supplementary motor area		8.04	3	−21	69
L. paracentral lobule		7.81	−3	−36	66
R. cerebellum anterior lobe	378	9.59	18	−39	−27
L. cerebellum anterior lobe		8.64	−15	−39	−27
R. inferior parietal lobule	18	4.49	48	−30	21
**Real machine > imagined machine**					
R. cerebellum anterior lobe	7796	13.59	18	−36	−27
L. cerebellum anterior lobe		12.45	−21	−36	−30
R. postcentral gyrus	508	10.07	15	−45	78
L. precuneus		8.58	−12	−51	78
R. superior parietal gyrus		6.64	21	−54	75
R. supplementary motor area		5.99	9	−9	78
L. paracentral lobule		5.68	−6	−27	78

*P_*FDR*_ < 0.025, voxels > 10. L indicates left; R indicates right.*

**TABLE 2:** Neural responses to combined manual massage andcombined machine massage under PLC.

Brain region	Voxels	Peak-*t* value	*x*	*y*	*z*
**Combined social > rest control**					
L. paracentral lobule	1350	12.06	−3	−36	63
R. postcentral gyrus		11.00	18	−42	69
L. paracentral lobule		10.83	−12	−39	72
R. supplementary motor area		8.75	6	−21	66
L. inferior parietal lobule		5.43	−42	−45	60
L. cerebellum anterior lobe	441	9.99	−15	−39	−27
R. cerebellum anterior lobe		8.50	18	−39	−27
L. precentral gyrus/supramarginal gyrus	18	4.39	−60	−21	42
L. superior temporal sulcus	16	4.39	−60	3	3
R. postcentral gyrus	30	4.10	54	−21	39
L. lingual gyrus	22	3.84	−12	−93	−12
**Combined machine > rest control**					
R. postcentral gyrus	2026	10.37	12	−33	75
L. precuneus		9.05	−9	−42	72
L. paracentral lobule		8.92	−9	−30	75
L. cerebellum anterior lobe	5336	10.02	−21	−33	−33
R. cerebellum anterior lobe		9.06	21	−33	−30
L. fusiform gyrus		5.97	−24	0	−39
L. cerebellum	13	3.20	−24	−81	−21
**Combined social > combined machine**					
L. middle frontal gyrus	24375	6.56	−27	54	27
L. middle frontal gyrus		5.94	−48	42	21
R. dorsal lateral prefrontal cortex		5.70	3	57	27
L. anterior cingulate cortex		4.94	−6	30	21
L. anterior insula		3.75	−33	6	15
L. orbitofrontal cortex		3.69	−9	36	−24
R. superior temporal sulcus		3.19	42	−45	15
R. cerebellum	17	4.52	36	−87	−42

*P_*FDR*_ < 0.025, voxels > 10. L indicates left; R indicates right.*

### The Effects of Intranasal OT on Behavioral and Neural Responses to Massage

**FIGURE 3:**
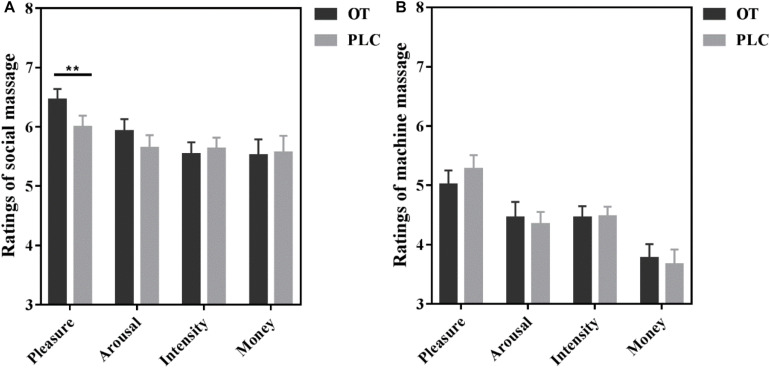
Behavioral ratings of **(A)** manually administered massage and **(B)** machine administered massage under oxytocin (OT) and placebo (PLC). Histograms show mean ± SEM rating scores for pleasantness, arousal, intensity and payment willingness for each massage condition. Error bars show standard errors. **p < 0.01.

Repeated-measure analysis of variance (ANOVA) of rating scores for manual and machine massage revealed a significant main effect of massage type [*F*(1,45) = 21.50, *p* < 0.001, η^2^ = 0.32] with subjects rating manual massage (6.23 ± 0.16) more pleasant than machine massage (5.15 ± 0.20) (*p* < 0.001) and a significant interaction between massage type and treatment [*F*(1,45) = 5.81, *p* = 0.02, η^2^ = 0.11]. The *post hoc* Bonferroni corrected analysis showed that OT significantly increased pleasantness ratings for manual (*p* = 0.008) but not for machine (*p* = 0.220) massage. No significant effects of OT were found for arousal (all *p*s > 0.265) or intensity (all *p*s > 0.759) ratings, or for ratings of how much subjects were willing to pay (all *p*s > 0.667) for either manual or machine massage (see Figure 3). Thus, OT selectively increased pleasantness ratings for manually administered massage compared with PLC.

OT administration had no significant effect on subjects’ perception of the gender of the person giving the massage (28 males vs. 18 females under OT compared with 24 males vs. 22 females under PLC) (Chi square = 0.71, *p* = 0.40). Overall, effects of OT on increased pleasantness ratings were similar irrespective of whether subjects thought the massage was administered by a male or female (mean ± SEM for male: PLC = 5.75 ± 0.29 and OT 6.32 ± 0.25; for female: PLC = 6.27 ± 0.22 and OT = 6.67 ± 0.23).

**FIGURE 4:**
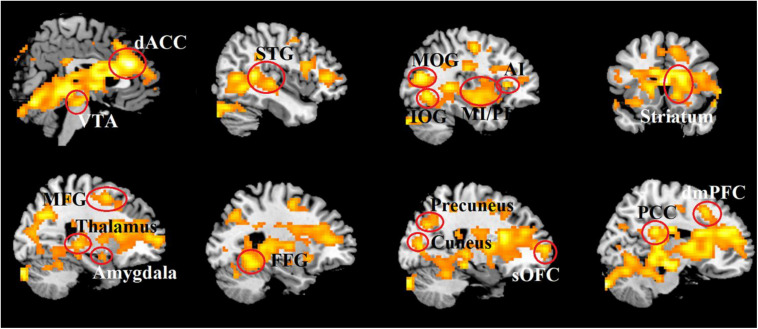
Increased brain activity induced by OT in response to combined real and imagined manually administered massage. Statistical maps are displayed with a threshold of *P* < 0.025, FDR corrected.

At the neural level, the whole brain analysis revealed that OT relative to PLC treatment increased responses in an extensive network of regions during combined real and imagined manual massage, but not to machine massage. These included brain reward (the OFC, dorsal striatum and ventral tegmental area), salience (the dACC and anterior and posterior insula), default mode (the mPFC, posterior cingulate cortex, precuneus and parahippocampal gyrus), emotion and memory (amygdala, hippocampus), social cognition (the superior and medial temporal regions and inferior parietal lobule) and visual (cuneus, fusiform gyrus and occipital gyrus) and somatosensory/motor processing regions (post-central gyrus, cerebellum) (see Table 3 and Figure 4).

**TABLE 3:** Significant effects of OT on neural activations inresponse to combined real and imagined massage by hand atwhole brain level.

Brain region	voxels	Peak-*t* value	*x*	*y*	*z*
R. dorsal anterior cingulate cortex	13644	5.09	3	33	24
R. cerebellum anterior lobe		4.71	6	−42	−6
L. inferior frontal gyrus		4.43	−54	36	3
L. inferior parietal lobule		4.36	−45	−36	27
R. superior temporal sulcus		4.32	42	−45	9
R. dorsal striatum		4.31	21	15	18
L. middle occipital gyrus		4.28	−36	−72	15
L. dorsal striatum		4.26	−21	−9	6
L. thalamus		4.21	−12	−6	6
R. midbrain/ventral tegmentum		4.20	12	−18	−12
L. precuneus		4.17	−21	−69	39
L. thalamus		4.13	−18	−18	12
R. dorsal striatum		4.10	15	3	9
L. midbrain/ventral tegmentum		4.09	−12	−21	−9
R. posterior cingulate cortex		4.08	12	−39	27
L. inferior occipital gyrus		4.08	−39	−63	−6
L. superior temporal sulcus		4.07	−39	−39	3
R. precuneus		4.01	27	−63	33
R. inferior orbital frontal cortex		3.90	57	21	−6
R. hippocampus		3.85	33	−36	−3
L. fusiform gyrus		3.84	−27	−51	−15
R. medial prefrontal gyrus		3.80	9	51	15
R. middle frontal gyrus		3.76	39	39	9
R. superior orbital frontal cortex		3.76	21	60	6
R. superior temporal sulcus		3.68	63	6	−6
R. dorsal striatum		3.68	15	18	0
R. middle frontal gyrus		3.63	33	−6	48
R. dorsal medial prefrontal gyrus		3.61	12	18	48
L. anterior insula		3.66	−36	24	6
L. middle frontal gyrus		3.51	−27	−6	51
L. postcentral gyrus		3.51	−36	−18	27
L. posterior insula		3.48	−36	−18	−3
L. middle insula		3.39	−36	3	−3
R. fusiform gyrus		3.32	33	−54	−18
L. cuneus		3.26	−18	−87	3
R. middle temporal gyrus		3.23	57	−15	−6
L. middle temporal gyrus		3.22	−51	−60	3
R. amygdala		3.07	24	−3	−15
L. precentral gyrus	216	3.79	−39	−12	51
L. middle frontal gyrus		3.51	−27	−6	51
R. postcentral gyrus	55	3.42	21	−42	51

*All with a P_*FDR*_ < 0.025 threshold and cluster > 10 voxels. L indicates left; R indicates right.*

### Correlations Between Neural Responses to Manual Massage and Behavioral Ratings, Trait Autism and Basal Plasma OT Concentrations

Under PLC, there were significant correlations between neural responses and pleasantness ratings for combined real and imagined manual massage for the left posterior insula (*r* = 0.30, *p* = 0.04), right amygdala (*r* = 0.38, *p* = 0.009), right fusiform gyrus (*r* = 0.37, *p* = 0.01) and right medial temporal gyrus (*r* = 0.31, *p* = 0.03). This positive correlation with pleasantness ratings was also found for the right amygdala under OT (*r* = 0.32, *p* = 0.03). There were also significant positive associations between neural activity and how much subjects rated they were prepared to pay for the manual massage under PLC in regions including the left posterior insula (*r* = 0.32, *p* = 0.03), left AI (*r* = 0.30, *p* = 0.04) and right dorsal striatum (*r* = 0.29, *p* = 0.05). Moreover, basal OT concentrations (mean ± SEM: 15.59 ± 0.96 pg/ml) were significantly negatively associated with the right hippocampus activity under PLC (*r* = −0.31, *p* = 0.04) and the autistic trait score (Total ASQ, *r* = 0.373, *p* = 0.011).

Given the within-subject design, correlations of treatment effects were tested using difference scores between OT and PLC treatments (i.e., OT-PLC). At the behavioral level, the treatment difference of amount of money subjects were willing to pay for the manual massage was found to be negatively correlated with the total ASQ scores (*r* = −0.30, *p* = 0.047). At the neural level, for the left precuneus there was a positive correlation with the total ASQ score (*r* = 0.31, *p* = 0.04), indicating that individuals with higher trait autism showed greater effects of OT administration.

Results also revealed that individual sensory responsivity modulated OT’s enhanced effects on neural responses to manual massage as the brain activity difference in cerebellum (*r* = −0.32, *p* = 0.03) and thalamus (*r* = −0.31, *p* = 0.04) was significantly negatively correlated with total scores of the SensOR Scales and the treatment difference of neural response in right STS (*r* = −0.38, *p* = 0.01), left (*r* = −0.35, *p* = 0.02) and right dorsal striatum (*r* = −0.34, *p* = 0.02) to manual massage also negatively associated with the scores of the tactile subscale of the SensOR Scales. Additionally, correlation analysis showed that the treatment difference in response to manual massage in the right ventral tegmental area (*r* = 0.31, *p* = 0.03), right cerebellum (*r* = 0.30, *p* = 0.03), right hippocampus (*r* = 0.34, *p* = 0.03), and right post-central gyrus (*r* = 0.32, *p* = 0.03) was positively correlated with basal OT concentrations. No other significant differences were observed for correlation measures between neural response to manual massage and behavioral ratings, autism trait or social touch scores or basal OT concentrations.

## Discussion

In the current study, we investigated whether intranasal OT modulates behavioral and neural responses to social touch administered as manually administered foot massage as opposed to impersonal machine-administered massage. Subjects underwent blocks of massage of each type and during the task they received the real massage and subsequently also imagined it. Under PLC, at the whole brain level manual massage evoked extensive activation changes in brain regions associated with affective touch processing, reward and social cognition as well as motor control regions. Similar patterns of activation changes occurred during imagined manual massage with the exception of an absence of activations in motor control regions (postcentral gyrus and cerebellum). The same general difference was observed in responses to imagined compared with real machine massage. Manual massage produced significantly greater responses than machine massage in regions particularly associated with processing social touch (including the OFC, ACC, insula, and STS). As hypothesized, intranasal OT significantly increased subjective pleasantness ratings of the manual but not machine massage. At the whole brain level OT evoked extensive increased neural responses to combined real and imagined manual massage, but not to machine massage, in key regions involved in reward (OFC, dorsal striatum and ventral tegmental area), social cognition (STS and inferior parietal lobule) and emotional and salience (amygdala, ACC and insula), visual (fusiform gyrus and occipital cortex), auditory (medial temporal gyrus) processing as well as in the default mode network (mPFC, parahippocampal gyrus, posterior cingulate and precuneus) and some sensorimotor regions (cerebellum and pre- and post-central gyrus). Altered activation of the amygdala in both OT and PLC treatment conditions showed positive association with pleasantness ratings. There were relatively few significant associations between trait autism and neural responses to manual massage (only precuneus) but we confirmed our previous findings (Li et al., 2019) for a negative association between autistic traits and basal plasma concentrations of OT.

### Behavioral and Neural Responses to Manual and Machine Administered Massage Under PLC

Under PLC, subjects rated machine administered massage as significantly less pleasurable and were willing to pay less for it, which was not significantly influenced by whether they thought the masseur was a female or a male. This replicated our findings from a previous experiment in Chinese male subjects (Li et al., 2019) but was in contrast to observations in male Caucasian subjects responding to social affective touch where pleasure ratings were much lower if they thought a man was touching them (Scheele et al., 2014a; Ellingsen et al., 2016). This presumably reflects cultural differences since massage is routinely administered by both males and females in China and all our subjects indicated prior to the experiment that they were happy to be massaged by either a male or a female.

At the neural level, we first examined whether imagined massage was as effective as real massage and found that patterns of neural responses when subjects simply imagined receiving either manual or machine administered foot massage differed significantly from real massage at the whole brain level only in two main motor control regions, the post-central gyrus and cerebellum under PLC. This emphasized both how good subjects were at imagining being massaged and that stronger activation of motor control regions during real massage should mainly reflect movement of the foot and leg due to the massage force being exerted on them. Our finding is consistent with a growing number of studies supporting Lang’s Bio-informational theory which demonstrated that similar emotional and neural responses occur during imagined and real interactions with emotional stimuli (Kim et al., 2007; Costa et al., 2010; Lewis et al., 2013; Ji et al., 2016). Further support can also be found in a more recent single neuron recording case-study in humans reporting that parietal cortex neurons can respond equivalently to both real and imagined touch (Chivukula et al., 2020). Mental imagery has also been used in clinical contexts as a means of facilitating behavioral fear extinction combined with exposure therapy (see Ji et al., 2016; Mertens et al., 2020) and recovery of motor control in Parkinson’s disease (Caligiore et al., 2017). Based on previous findings that massage, including the ones applied on foot, can facilitate endogenous OT release (Morhenn et al., 2012; Li et al., 2019), our current pilot findings that imagined massage evoked similar responses to real massage suggest that it may be a potential future therapeutic strategy for inducing changes in neural systems responding to social touch and evoking associated OT release. Furthermore, hypnosis can facilitate sensory imagery perhaps by reducing inhibitory control (Case et al., 2015) and the combination of hypnosis and imagery has been widely used in immune control (Gruzelier, 2002), surgical anxiety reduction (Hammond, 2010; Vagnoli et al., 2019), postoperative recovery (Lambert, 1996) and motor training (Torem, 1992; Newmark and Bogacki, 2005). Thus a combination of imagined massage and hypnosis may be a more promising strategy in clinical application, although future studies are needed to uncover the detailed behavioral effects and the underlying neural substrates.

To increase statistical power, we therefore combined the real and imagined massage conditions in the following analyses. Combined real and imagined manually administered massage evoked extensive responses in a number of brain regions associated with positive affective touch (see Gordon et al., 2013; Kaiser et al., 2016; Sailer et al., 2016), including those involved in sensorimotor (cerebellum, paracentral lobule, postcentral gyrus and supplementary motor area), social cognition (inferior parietal lobule and STS) and visual processing (lingual gyrus). On the other hand, combined real and imagined machine massage primarily increased responses in sensorimotor processing regions. Contrary to expectation under PLC combined manual massage did not produce significantly increased responses in brain reward or salience processing regions. This is in line with a previous study reporting effects of Swedish foot massage (Sliz et al., 2012) although it contrasts with our previous ROI-based fNIRS study (Li et al., 2019), which may reflect the fact that subjects found massage in the MRI scanner environment less pleasurable than in a quiet comfortable room. Furthermore, in contrast to machine massage, manual massage produced significantly greater activation in regions of the affective touch networks involved in rewarding and salience aspects of massage (the OFC, ACC, and AI) as well as in social cognition (the STS). This is also in accordance with the behavioral findings that subjects found manual massage significantly more pleasant, arousing and intense than machine massage and were willing to pay more for it. In general agreement with Li et al. (2019), the stronger STS activation may reflect the more social nature of manual massage and indicates a potential psychological influence. Posterior superior temporal region responses are predictive of the pleasantness of stroking the skin (Davidovic et al., 2016) and tactile stimulation which does not involve being stroked by another person (i.e., administered using some kind of device) does not activate the posterior STS (Sliz et al., 2012; Perini and Olausson, 2015).

It should be noted that although we tried to make the manual and machine massages similar it is possible that differences between them could have contributed to our current findings. While in the current study when subjects received the machine massage they rated its intensity less and were less aroused than with the manual massage, this is unlikely to have had a major influence on observed differences in neural responses since in our previous fNIRS study where intensity and arousal effects of the different massages were equivalent we also found similar differences (Li et al., 2019). The machine massage protocol primarily applied rhythmic pressure to the foot which would be expected to target both CT- and A-β fibers, however, the manual massage additionally involved skin to skin contact and probably greater stimulation of CT-fibers as a result of touching even though the skin was not deliberately caressed. Some support for such a differential stimulation of CT-fibers by the different types of massage is indicated by our previous findings that both manual and machine administered massage evoked OT-release although that following manual massage was significantly greater (Li et al., 2019). It has been proposed that OT-release is primarily evoked following stimulation of CT-fibers (Uvnäs-Moberg et al., 2015; Walker et al., 2017). Thus, it is possible that other forms of machine administered massage which can stimulate CT-fibers more potently might produce neural and behavioral responses more akin to those observed following manual massage.

### Effects of Intranasal OT on Behavioral and Neural Responses to Massage

In line with previous social/affective touch studies (Scheele et al., 2014a; Chen et al., 2020), we found that intranasal OT administration increased ratings of pleasantness for manual massage. However, in contrast to Scheele et al. (2014a), but in line with those in our previous study (Li et al., 2019), we found no evidence for any modulatory effect of what sex the subjects thought the masseur was, which may reflect cultural differences as already discussed. There were no effects of OT on subjects’ arousal or intensity ratings for manual massage or how much they would pay for it and no effects on any behavioral ratings for machine administered massage. Subjects also reported a significant increase in their positive mood score after receiving the manual massage under OT but not PLC, which further supports the observation that intranasal OT appears to enhance the perceived pleasantness of manual massage. Another previous study has failed to find any effect of intranasal OT on the perceived pleasantness of short periods of light touch on the arm administered by either a person or a machine, which could be due to confounding effects from simultaneously being presented with emotional face stimuli (Ellingsen et al., 2014).

Intranasal OT increased responses to combined real and imagined manual massage in a widely distributed network, including all the regions previously reported to respond to social touch (Rolls et al., 2003; Rolls, 2010; Gordon et al., 2013; Ellingsen et al., 2016; Kaiser et al., 2016). This was in marked contrast to combined real and imagined machine massage where it produced no significant changes at all at the whole brain level. Not only were neural responses significantly increased in the OFC, AI and ACC, in line with a previous study on Caucasian males touched on their leg by an unseen female (Scheele et al., 2014a) and a study on Chinese males touched on their arm (Chen et al., 2020), but also in a far more extensive network of regions involved in attention, emotion, reward, salience, social cognition, sensorimotor and visual processing.

In addition to enhanced responses in the orbitofrontal reward network, we also found that OT increased activation in the dorsal striatum and ventral tegmental reward regions similar to previous studies reporting this in the context of facilitated learning during social feedback (Hu et al., 2015), increased responses to social reward (Groppe et al., 2013) and increased attractiveness ratings for faces of romantic partners (Scheele et al., 2013). The dorsal ACC and posterior insula also showed increased responses to combined manual massage following OT, in agreement with a previous study (Scheele et al., 2014a). The posterior insula is the main recipient of inputs from skin CT fibers conveying signals from affective touch stimulation (Olausson et al., 2002; McGlone et al., 2014) and thus OT appears to be increasing the response to these signals at an early stage of brain processing. As a core hub of the salience network (Uddin, 2015; Uddin et al., 2017) and emotion processing (Craig, 2009; Kurth et al., 2010; Lamm and Singer, 2010; Koban and Pourtois, 2014), the insula has been shown to involve the modulatory effects of OT on social salience processing in a number of social tasks (Riem et al., 2011; Striepens et al., 2012; Yao et al., 2018a, b). The dorsal ACC is also a key component of the salience network which responds to affective touch (Gordon et al., 2013) and may encode pleasant skin to skin touch (Rolls et al., 2003; Lindgren et al., 2012).

As the ACC, insula and OFC are functionally connected (Cauda et al., 2011), OT may be influencing this circuitry to produce a heightened sense of both the salience of the manual massage experience and self-awareness of its effects. Indeed, OT increased responses to manual massage in parts of the default mode network (mPFC, precuneus and posterior cingulate cortex) which are involved in self-consciousness and self-referential processing and thereby contribute to heightened self-awareness. Several previous studies have reported that intranasal OT administration can reduce self-awareness with reduced activation in the medial frontal cortex (Zhao et al., 2016, 2017) and focus on interoceptive cues in favor of greater awareness of others or salient external social cues when altering responses in the AI and its coupling with the posterior insula (Yao et al., 2018a). On the other hand, OT can also increase selfish behavior in other contexts with no immediate external distractors (Scheele et al., 2014b; Xu et al., 2019; Sindermann et al., 2020). Thus, in the context of receiving manual massage by an unseen masseur, and where there are therefore no salient external social cues, it may serve to heighten both awareness of self and interoceptive orientation and the resultant level of pleasure experienced.

The amygdala also exhibited increased responses to combined real and imagined manual massage following OT treatment and its activation was positively associated with pleasantness ratings under both OT and PLC. A previous study has reported that pleasant touch targeting CT fibers can activate the amygdala (Voos et al., 2013) and the amygdala is one of the key brain regions modulated by intranasal OT treatment (see Kendrick et al., 2017). While much of the focus on OT modulation of amygdala responses has been in the context of negative emotional stimuli (Kendrick et al., 2017), it can also enhance its responses to positive social ones (Gamer et al., 2010; Gao et al., 2016). Thus in the context of pleasant touch, the increased amygdala activity may reflect a positive emotional representation associated with enhanced pleasantness.

The superior temporal region and inferior parietal lobule are key components of the mirror neuron system as well as for social cognition, therefore the finding that OT enhances their response to combined manual massage together with in frontal (inferior frontal gyrus and insula) and sensorimotor components (pre- and post-central gyrus) of the mirror neuron system indicates a mechanism whereby it could modulate empathy, imitation, language, self-other distinction and theory of mind (Keysers et al., 2018). Furthermore, components of the mirror neuron system influencing these behaviors have been reported to be dysfunctional in both autism spectrum disorder and schizophrenia (see Minichino and Cadenhead, 2017).

Intranasal OT also increased responses to massage in a number of primary and association auditory and visual cortical processing regions as well as the thalamus. There is increasing evidence for integration between visual and tactile processing in the brain, with studies reporting that tactile stimulation evokes responses in visual processing regions (see Lacey and Sathian, 2016), which may be important for multisensory object recognition and reflect touch-evoked complimentary visual imagery. Supporting evidence for this visuo-tactile integration is provided by the “rubber hand illusion” (see Lacey and Sathian, 2016) with autistic individuals being less susceptible to this illusion implying impaired visuo-tactile integration (Cascio et al., 2012), and concentrations of OT in saliva being correlated with the strength of this illusion in healthy subjects (Ide and Wada, 2017).

### Associations Between Behavioral and Neural Responses to Massage and Autistic Traits and Basal OT Concentrations

In the current study, unlike our previous fNIRS study (Li et al., 2019) and an intranasal OT study (Scheele et al., 2014a), we did not find any association between OFC responses to manual massage and trait autism or STQ scores either under PLC or OT. Decreased OFC activation in autistic subjects has been reported in response to affective touch (Kaiser et al., 2016), as well as in healthy subjects with higher trait autism (Voos et al., 2013). However, we did observe that the difference in activation of the right precuneus between OT and PLC treatments was positively associated with ASQ scores, indicating that this region which is important for self-other distinction (Cabanis et al., 2013; Zhao et al., 2016) was more strongly influenced by OT in individuals with higher trait autism during manual massage.

Manual massage evoked responses in both the insula and other brain reward regions such as the dorsal striatum were significantly associated with either pleasure ratings and/or with how much subjects were willing to pay for the massage, indicating that these regions are closely associated with the rewarding value of massage. However, no significant associations were found between behavioral responses to manual massage and ASQ scores and basal OT concentrations in the current study, although we confirmed our observation in the previous study (Li et al., 2019) that they were negatively associated with the ASQ scores. This inconsistency may be due to differences between perceived massage pleasantness in the MRI scanner environment and in a quiet comfortable room as already discussed.

As in our previous study (Li et al., 2019) we found no associations between basal plasma OT concentrations and pleasantness ratings. In the current study for neural responses to combined manual massage there was a significant positive association between basal OT concentrations and the increased effects of OT on activation in the right ventral tegmentum, hippocampus, post-central gyrus and cerebellum. This provides some evidence that reward and sensorimotor effects of intranasal OT may be strongest in individuals with higher basal plasma concentrations, although it should be emphasized that the majority of regions responding to OT did not show this association. We were unable to take blood samples from subjects in the MRI scanner to assess massage-evoked changes following OT or PLC treatment to assess possible correlations with behavioral or neural changes. However, in our previous fNIRS study where we could do this we found no significant associations and so it is likely the same would have been the case in the current study. This may possibly reflect the fact that OT concentrations are measured at a single time point whereas massage is administered over a long period and/or that increases in OT influence neural responses to massage indirectly via modulation of classical transmitters or other peptides (see Kendrick, 2000).

It should be noted that we used an established ELISA to quantify OT concentrations in plasma and which incorporated the recommended extraction step as well as confirming the accuracy of measures by incorporating sampled spiked with known OT concentrations. However, there is still some debate concerning precisely what is measured in such assays other than OT itself although there is generally reasonable concordance between radioimmunoassay (RIA) and ELISAs in this respect following extraction (McCullough et al., 2013; Leng and Sabatier, 2016). Our basal OT concentrations using the Cayman ELISA were slightly higher (mean 15.59 pg/ml) than many, but not all, reported in studies using RIA and also in some ELISAs following extraction (Szeto et al., 2011). They are also similar to those reported in a study on both mothers and fathers prior to skin to skin contact with infants and also using an RIA (Velandia, 2012). Furthermore, they are equivalent to those we have reported previously (Li et al., 2019; Chen et al., 2020) and also from another independent group on Chinese subjects with the more extensively used ENZO ELISA following extraction (Zhang et al., 2016). We cannot, however, entirely rule out the possibility that the antibodies in the assays were also sensitive to some OT fragments although this is a potential issue for all types of OT assays. The subjects were clearly not unduly stressed by the routine venipuncture procedures and basal concentrations were similar in the current study following venipuncture as in our previous one where indwelling catheters were used (Li et al., 2019).

### Potential Mechanisms Involved in Intranasal OT-Evoked Facilitation of Responses to Manual Massage

Although there has been some debate about whether intranasally-applied OT reaches the brain (Leng and Ludwig, 2016) accumulating evidence, primarily from animal models, has demonstrated that it can both enter the brain directly, possibly via olfactory and trigeminal nerves, and indirectly by binding to the receptor for advanced glycation end-products (RAGE) and being transported across the blood brain barrier (Yamamoto and Higashida, 2020). Both of these direct and indirect routes of exogenous OT administration can lead to wide ranging changes in neural activity although there may be some regional and functional differences (Ferris et al., 2015; Quintana et al., 2016; Dumais et al., 2017; Tanaka et al., 2018; Martins et al., 2020). A third potential indirect route is via vagal stimulation following effects on receptors in peripheral organs such as the heart and gastrointestinal system (see Quintana et al., 2020 for recent review). An additional potential variable is whether OT itself is having functional effects on responses to touch or if various fragments of the peptide following degradation or derived from other sources are responsible via actions on receptors other than the OT receptor and which might more readily cross the blood brain barrier (see Uvnäs-Moberg et al., 2019).

The majority of brain regions exhibiting enhanced responses to manual massage following OT administration express OT receptors in humans (see Quintana et al., 2018) and thus it is possible that increased cerebrospinal fluid concentrations of OT following intranasal administration (see Striepens et al., 2013) could act on these receptors in a paracrine manner to potentiate massage-induced effects. Exogenously administered OT could also exert a positive feedback effect via hypothalamic autoreceptors (see Kendrick, 2000) to potentiate massage-induced OT release thereby increasing activation in regions receiving hypothalamic projections. Indeed, direct infusions of OT into the paraventricular nucleus of sheep can stimulate both maternal behavior and bonding in sheep (Da Costa et al., 1996). Although intranasal OT does not appear to influence basal endogenous concentrations (Lee et al., 2018), it is still possible that it might enhance stimulus-evoked ones. Depending on its intensity, massage can also influence either sympathetic or parasympathetic nervous system responses via skin pressure receptors (Field, 2010, 2019) which raises the additional possibility that OT is influencing neural responses via facilitating vagal responses to pressure receptor activation (A-β fibers) as well as via CT-fibers. Indeed, previous studies have reported that OT can influence both parasympathetic (Kemp et al., 2012) and sympathetic (De Oliveira et al., 2012) nervous system activity. However, as discussed above we would anticipate that pressure-induced activation of A-β fibers should be broadly similar with both manual and machine administered massage while CT-fiber activation is likely to have been stronger during the manually administered massage. Finally, recent studies have also demonstrated the presence of OT receptors in cutaneous nociceptive fibers (C- and A-δ but not A-β fibers) in rats via which OT may exert analgesic effects (González-Hernández et al., 2017). Exogenously administered OT might therefore act to alter the sensitivity of skin to touch via these receptors. This possibility receives some support from findings that post-partum women who have had babies delivered by cesarean section require a subsequent infusion of OT in order for them to respond equivalently to skin to skin contact with their babies as women undergoing normal delivery in terms of OT release and also psychological adaptations (e.g., reduced anxiety and aggression and increased social interactive skills) (Velandia, 2012; Uvnäs-Moberg et al., 2020). However, although we did not measure subjects’ sensitivity to massage directly in the present study there were no significant rating changes of either massage intensity or associated arousal following either manually or machine administered massage which might have been expected if OT had influenced skin sensitivity. In our previous study showing OT-facilitated effects on neural responses and pleasantness ratings following touch on the arm administered using different materials there were similarly no effects on intensity or arousal ratings (Chen et al., 2020). Additionally, in the Scheele et al. (2014a) study OT-effects on enhancing pleasantness and neural responses to rhythmic caressing touch to the leg only occurred when subjects thought it was a female but not male touching them (even though it was always the same female touching them), suggesting OT was mainly having centrally-mediated effects influencing psychological factors. At this stage, however, we have insufficient information to reliably disentangle these various potential peripheral and centrally-mediated mechanisms of OT action.

### Limitations

We acknowledge several limitations in our current study. Firstly, we only included male subjects as in previous studies investigating effects of OT on touch. There is increasing evidence that OT can have sex dependent effects (Gao et al., 2016; Luo et al., 2017; Ma et al., 2018) and so it is possible that OT might have different effects on responses to massage in females. Secondly, experiencing short periods of massage in a controlled manner inside an MRI scanner is somewhat artificial and even potentially stressful so it would be important to demonstrate beneficial effects of intranasal OT in a more normal and comfortable environment. Thirdly, given the paradigm and technique used in the present study, we cannot specify which nerve fibers are activated by massage. While the CT fibers have been found to be more closely associated with social affective touch (Gordon et al., 2013; Ackerley et al., 2014; McGlone et al., 2014; Pawling et al., 2017), Field (2019) suggests that massage therapy with deeper pressure may activate A-β afferents. It is likely that massage administered with rhythmic pressure as used in the present study may activate both the CT and A-β fibers. Future studies are needed to investigate the specific mechanisms underlying nerve fiber afferents activated by different forms of tactile stimulation. Fourthly, observed differences between manual and machine massage may have been contributed to some extent by the manual massage stimulating CT-fibers to a greater extent. Finally, in our paradigm real and imagined massage were performed sequentially during scanning in order to make it easier for subjects to imagine each type of massage. Although we identified major differences only in motor regions between the effects of real and imagined massage using this paradigm, it is possible that if real and imagined massage periods were given as independent blocks this might have revealed greater differences and possibly also some differential effects of OT.

### Summary

Taken together our findings demonstrate that intranasal OT can facilitate the positive neural and behavioral effects of real and imagined massage administered manually and influence many of the brain regions and circuits which are dysfunctional in autism. This supports the possible therapeutic combination of intranasal OT and massage in autistic individuals.

## Data Availability Statement

The raw data supporting the conclusions of this article will be made available by the authors, without undue reservation, to any qualified researcher.

## Ethics Statement

The studies involving human participants were reviewed and approved by the local ethics committee at the University of Electronic Science and Technology of China. The patients/participants provided their written informed consent to participate in this study.

## Author Contributions

YC, SY, QL, and KK designed this study. YC, QZ, and HC collected the data. YC, SY, and JK conducted the statistical analysis. YZ and JW analyzed the plasma oxytocin levels. YC prepared the manuscript. YC, KK, SY, BB, and CM revised the manuscript into its finalized form. All authors contributed to the article and approved the submitted version.

## Conflict of Interest

The authors declare that the research was conducted in the absence of any commercial or financial relationships that could be construed as a potential conflict of interest.

## Footnotes

1 http://www.fil.ion.ucl.ac.uk/spm/spm12

## References

[B1] Ackerley R. Saar K. McGlone F. Backlund Wasling H. (2014). Quantifying the sensory and emotional perception of touch: differences between glabrous and hairy skin. *Front. Behav. Neurosci.* 8:34. 10.1523/JNEUROSCI.2847-13.2014 24574985 PMC3920190

[B2] Agren G. Lundeberg T. Uvnäs-Moberg K. Sato A. (1995). The oxytocin antagonist 1-deamino-2-D-Tyr-(Oet)-4-Thr-8-Orn-oxytocin reverses the increase in the withdrawal response latency to thermal, but not mechanical nociceptive stimuli following oxytocin administration or massage-like stroking in rats. *Neurosci. Lett.* 187 49–52. 10.1016/0304-3940(95)11335-T 7617300

[B3] Baron-Cohen S. Wheelwright S. (2004). The empathy quotient: an investigation of adults with Asperger syndrome or high functioning autism, and normal sex differences. *J. Autism Dev. Disord.* 34 163–175. 10.1023/b:jadd.0000022607.19833.00 15162935

[B4] Baron-Cohen S. Wheelwright S. Skinner R. Martin J. Clubley E. (2001). The autism-spectrum quotient (AQ): evidence from asperger syndrome/high-functioning autism, males and females, scientists and mathematicians. *J. Autism Dev. Disord.* 31 5–17. 10.1023/a:1005653411471 11439754

[B5] Bartz J. A. Zaki J. Bolger N. Ochsner K. N. (2011). Social effects of oxytocin in humans: context and person matter. *Trends Cogn. Sci.* 15 301–309. 10.1016/j.tics.2011.05.002 21696997

[B6] Beck A. T. Steer R. A. Brown G. K. (1996). *Manual for the Beck Depression Inventory-II.* San Antonio, TX: Psychol. Corp.

[B7] Björnsdotter M. Gordon I. Pelphrey K. A. Olausson H. Kaiser M. (2014). Development of brain mechanisms for processing affective touch. *Front. Behav. Neurosci.* 8:24. 10.3389/fnbeh.2014.00024 24550800 PMC3912430

[B8] Björnsdotter M. Morrison I. Olausson H. (2010). Feeling good: on the role of C fiber mediated touch in interoception. *Exp. Brain. Res.* 207 149–155. 10.1007/s00221-010-2408-y 20963582

[B9] Cabanis M. Pyka M. Mehl S. Müller B. W. Loos-Jankowiak S. Winterer G. (2013). The precuneus and the insula in self-attributional processes. *Cogn. Affect. Behav. Neurosci.* 13 330–345. 10.3758/s13415-012-0143-5 23297009

[B10] Caligiore D. Mustile M. Spalletta G. Baldassarre G. (2017). Action observation and motor imagery for rehabilitation in Parkinson’s disease: a systematic review and an integrative hypothesis. *Neurosci. Biobehav. Rev.* 72 210–222. 10.1016/j.neubiorev.2016.11.005 27865800

[B11] Cascio C. J. Foss-Feig J. H. Burnette C. P. Heacock J. L. Cosby A. A. (2012). The rubber hand illusion in children with autism spectrum disorders: delayed influence of combined tactile and visual input on proprioception. *Autism* 16 406–419. 10.1177/1362361311430404 22399451 PMC3529180

[B12] Cascio C. J. Moore D. McGlone F. (2019). Social touch and human development. *Dev. Cogn. Neurosci.* 35 5–11. 10.1016/j.dcn.2018.04.009 29731417 PMC6968965

[B13] Case L. K. Pineda J. Ramachandran V. S. (2015). Common coding and dynamic interactions between observed, imagined, and experienced motor and somatosensory activity. *Neuropsychologia* 79 233–245. 10.1016/j.neuropsychologia.2015.04.005i 25863237 PMC4600026

[B14] Cauda F. D’Agata F. Sacco K. Duca S. Geminiani G. Vercelli A. (2011). Functional connectivity of the insula in the resting brain. *Neuroimage* 55 8–23. 10.1016/j.neuroimage.2010.11.049 21111053

[B15] Chen Y. Becker B. Zhang Y. Cui H. Du J. Wernicke J. (2020). Oxytocin increases the pleasantness of affective touch and orbitofrontal cortex activity independent of valence. *Eur. Neuropsychopharm* 39 99–110. 10.1016/2020.08.003 32861545

[B16] Chivukula S. Zhang C. Aflalo T. Jafari M. Pejsa K. Pouratian N. (2020). Neural encoding of felt and imagined touch within posterior parietal cortex. *bioRxiv* [Preprint]. 10.1101/2020.07.27.226633 PMC7924956 33647233

[B17] Costa V. D. Lang P. J. Sabatinelli D. Versace F. Bradley M. M. (2010). Emotional imagery: assessing pleasure and arousal in the brain’s reward circuitry. *Hum. Brain. Mapp.* 31 1446–1457. 10.1002/hbm.20948 20127869 PMC3620013

[B18] Craig A. D. (2009). How do you feel–now? The anterior insula and human awareness. *Nat. Rev. Neurosci.* 10 59–70. 10.1038/nrn2555 19096369

[B19] Crockford C. Wittig R. M. Langergraber K. Ziegler T. E. Zuberbuhler K. Deschner T. (2013). Urinary oxytocin and social bonding in related and unrelated wild chimpanzees. *Proc. Biol. Sci.* 280:20122765. 10.1098/rspb.2012.2765 23345575 PMC3574389

[B20] Croy I. Luong A. Triscoli C. Hofmann E. Olausson H. Sailer U. (2016). Interpersonal stroking touch is targeted to C tactile afferent activation. *Behav. Brain Res.* 297 37–40. 10.1016/j.bbr.2015.09.038 26433145

[B21] Da Costa A. P. C. Guevara-Guzman R. G. Ohkura S. Goode J. A. Kendrick K. M. (1996). The role of oxytocin release in the paraventricular nucleus in the control of maternal behavior in sheep. *J. Neuroendocrinol.* 8 163–177. 8730650 10.1046/j.1365-2826.1996.04411.x

[B22] Davidovic M. Jönsson E. H. Olausson H. Björnsdotter M. (2016). Posterior superior temporal sulcus responses predict perceived pleasantness of skin stroking. *Front. Hum. Neurosci.* 10:432. 10.3389/fnhum.2016.00432 27679564 PMC5020046

[B23] De Oliveira D. C. G. Zuardi A. W. Graeff F. G. Queiroz R. H. C. Crippa J. A. S. (2012). Anxiolytic-like effect of oxytocin in the simulated public speaking test. *J. Psychopharm.* 26 497–504. 10.1177/0269881111400642 21555332

[B24] Dumais K. M. Kulkarni P. P. Ferris C. F. Veenema A. H. (2017). Sex differences in neural activation following different routes of oxytocin administration in awake adult rats. *Psychoneuroendocrinology* 81 52–62. 10.1016/j.psyneuen.2017.04.003 28412582 PMC5497485

[B25] Dunbar R. I. (2010). The social role of touch in humans and primates: behavioural function and neurobiological mechanisms. *Neurosci. Biobehav. Rev.* 34 260–268. 10.1016/j.neubiorev.2008.07.001 18662717

[B26] Ellingsen D.-M. Leknes S. Løseth G. Wessberg J. Olausson H. (2016). The neurobiology shaping affective touch: expectation, motivation, and meaning in the multisensory context. *Front Psychol.* 6:1986. 10.3389/fpsyg.2015.01986 26779092 PMC4701942

[B27] Ellingsen D.-M. Wessberg J. Cheinokova O. Olausson H. Laeng B. Leknes S. (2014). In touch with your emotions: oxytocin and touch change social impressions while others’ facial expressions can alter touch. *Psychoneuroendocrinology* 39 11–20. 10.1016/j.psyneuen.2013.09.017 24275000

[B28] Espí-López G. V. Zurriaga-Llorens R. Monzani L. Falla D. (2016). The effect of manipulation plus massage therapy versus massage therapy alone in people with tension-type headache. A randomized controlled clinical trial. *Eur. J. Phys. Rehabi.l Med.* 52 606–617. 26989818

[B29] Essick G. K. McGlone F. Dancer C. Fabricant D. Ragin Y. Phillips N. (2010). Quantitative assessment of pleasant touch. *Neurosci. Biobehav. Rev.* 34 192–203. 10.1016/j.neubiorev.2009.02.003 19896001

[B30] Ferris C. F. Yee J. R. Kenkel W. M. Dumais K. M. Moore K. Veenema A. H. (2015). Distinct BOLD activation profiles following central and peripheral oxytocin administration in awake rats. *Front. Behav. Neurosci.* 9:245. 10.3389/fnbeh.2015.00245 26441574 PMC4585275

[B31] Field T. (2010). Touch for socioemotional and physical well-being: a review. *Dev. Rev.* 30 367–383. 10.1016/j.dr.2011.01.001

[B32] Field T. (2019). Pediatric massage therapy research: a narrative review. *Children* 6:78. 10.3390/children6060078 31174382 PMC6617372

[B33] Friston K. J. Holmes A. P. Worsley K. J. Poline J. P. Frith C. D. Frackowiak R. S. (1994). Statistical parametric maps in functional imaging: a general linear approach. *Hum. Brain Mapp.* 2 189–210.

[B34] Gamer M. Zurowski B. Büchel C. (2010). Different amygdala subregions mediate valence-related and attentional effects of oxytocin in humans. *Proc. Natl. Acad. Sci. U.S.A.* 107 9400–9405. 10.1073/pnas.1000985107 20421469 PMC2889107

[B35] Gao S. Becker B. Luo L. Geng Y. Zhao W. Yin Y. (2016). Oxytocin, the peptide that bonds the sexes also divides them. *Proc. Natl. Acad. Sci. U.S.A.* 113 7650–7654. 10.1073/pnas.1602620113 27325780 PMC4941426

[B36] González-Hernández A. Manzano-García A. Martínez-Lorenzana G. Tello-García I. A. Carranza M. Arámburo C. (2017). Peripheral oxytocin receptors inhibit the nociceptive input signal to spinal dorsal horn wide-dynamic-range neurons. *Pain* 158 2117–2128. 10.1097/j.pain.0000000000001024 28731982

[B37] Gordon I. Voos A. C. Bennett R. H. Bolling D. Z. Pelphrey K. A. Kaiser M. D. (2013). Brain mechanisms for processing affective touch. *Hum. Brain. Mapp.* 34 914–922. 10.1002/hbm.21480 22125232 PMC6869848

[B38] Groppe S. E. Gossen A. Rademacher L. Hahn A. Westphal L. Gründer G. (2013). Oxytocin influences processing of socially relevant cues in the ventral tegmental area of the human brain. *Biol. Psychiatry* 74 172–179. 10.1016/j.biopsych.2012.12.023 23419544

[B39] Gruzelier J. H. (2002). A review of the impact of hypnosis, relaxation, guided imagery and individual differences on aspects of immunity and health. *Stress* 5 147–163. 10.1080/10253890290027877 12186693

[B40] Hammond D. C. (2010). Hypnosis in the treatment of anxiety- and stress-related disorders. *Expert Rev. Neurother.* 10 263–273. 10.1586/ern.09.140 20136382

[B41] Heimberg R. G. Horner K. Juster H. Safren S. Brown E. Schneier F. (1999). Psychometric properties of the Liebowitz social anxiety scale. *Psychol. Med.* 29 199–212. 10.1017/s0033291798007879 10077308

[B42] Holt-Lunstad J. Birmingham W. A. Light K. C. (2008). Influence of a “warm touch support enhancement intervention among married couples on ambulatory blood pressure, oxytocin, alpha amylase, and cortisol. *Psychosom. Med.* 70 976–985. 10.1097/PSY.0b013e318187aef7 18842740

[B43] Hu J. Qi S. Becker B. Luo L. Gao S. Gong Q. (2015). Oxytocin selectively facilitates learning with social feedback and increases activity and functional connectivity in emotional memory and reward processing regions. *Hum. Brain. Mapp.* 36 2132–2146. 10.1002/hbm.22760 25664702 PMC6868957

[B44] Ide M. Wada M. (2017). Salivary oxytocin concentration associates with the subjective feeling of body ownership during the rubber hand illusion. *Front. Hum. Neurosci.* 11:166. 10.3389/fnhum.2017.00166 28439234 PMC5383663

[B45] Ji J. L. Heyes S. B. MacLeod C. Holmes E. A. (2016). Emotional mental imagery as simulation of reality: fear and beyond—A tribute to Peter Lang. *Behav. Ther.* 47 702–719. 10.1016/j.beth.2015.11.004 27816082 PMC5112008

[B46] Kaiser M. D. Yang D. Y.-J. Voos A. C. Bennett R. H. Gordon I. Pretzsch C. (2016). Brain mechanisms for processing affective (and nonaffective) touch are atypical in autism. *Cereb. Cortex.* 26 2705–2714. 10.1093/cercor/bhv125 26048952 PMC4869810

[B47] Kanner L. (1943). Autistic disturbances of affective contact. *Nervous Child.* 2 217–250. 4880460

[B48] Kemp A. H. Quintana D. S. Kuhnert R.-L. Griffiths K. Hickie I. B. Guastella A. J. (2012). Oxytocin increases heart rate variability in humans at rest: implications for social approach-related motivation and capacity for social engagement. *PLoS One* 7:e44014. 10.1371/journal.pone.0044014 22937145 PMC3429409

[B49] Kendrick K. M. (2000). Oxytocin, motherhood and bonding. *Exp. Physiol.* 85 111s–124s. 10.1111/j.1469-445x.2000.tb0004.x 10795913

[B50] Kendrick K. M. Guastella A. J. Becker B. (2017). “Overview of human oxytocin research,” in *Behavioral Pharmacology of Neuropeptides: Oxytocin*, eds Hurlemann R. Grinevich V. (Berlin: Springer), 321–348. 10.1007/7854_2017_19 28864976

[B51] Keysers C. Paracampo R. Gazzola V. (2018). What neuromodulation and lesion studies tell us about the function of the mirror neuron system and embodied cognition. *Curr. Opin. Psychol.* 24 35–40. 10.1016/j.copsyc.2018.04.001 29734039 PMC6173305

[B52] Kim S. E. Kim J. W. Kim J. J. Jeong B. S. Choi E. A. Jeong Y. G. (2007). The neural mechanism of imagining facial affective expression. *Brain Res.* 1145 128–137. 10.1016/j.brainres.2006.12.048 17359942

[B53] Koban L. Pourtois G. (2014). Brain systems underlying the affective and social monitoring of actions: an integrative review. *Neurosci. Biobehav. Rev.* 46 71–84. 10.1016/j.neubiorev.2014.02.014 24681006

[B54] Kreuder A. K. Scheele D. Wassermann L. Wollseifer M. Stoffel-Wagner B. Lee M. R. (2017). How the brain codes intimacy: the neurobiological substrates of romantic touch. *Hum. Brain. Mapp.* 38 4525–4534. 10.1002/hbm.23679 28580708 PMC6867116

[B55] Kurth F. Zilles K. Fox P. T. Laird A. R. Eickhoff S. B. (2010). A link between the systems: functional differentiation and integration within the human insula revealed by meta-analysis. *Brain Struct. Funct.* 214 519–534. 10.1007/s00429-010-0255-z 20512376 PMC4801482

[B56] Lacey S. Sathian K. (2016). “Crossmodal and multisensory interactions between vision and touch,” in *Scholarpedia of Touch. Scholarpedia*, eds Prescott T. Ahissar E. Izhikevich E. (Paris: Atlantis Press), 10.2991/978-94-6239-133-8_25 PMC4715428 26783412

[B57] Lambert S. A. (1996). The effects of hypnosis/guided imagery on the postoperative course of children. *J. Dev. Behav. Pediatr.* 17 307–310. 8897217 10.1097/00004703-199610000-00003

[B58] Lamm C. Singer T. (2010). The role of anterior insular cortex in social emotions. *Brain Struct. Funct.* 214 579–591. 10.1007/s00429-010-0251-3 20428887

[B59] Lang P. J. (1977). Imagery in therapy: an information processing analysis of fear. *Behav. Ther.* 8 862–886. 10.1016/S0005-7894(77)80157-3 27816081

[B60] Lang P. J. (1979). A bio-informational theory of emotional imagery. *Psychophysiology* 16 495–512. 10.1111/j.1469-8986.1979.tb01511.x 515293

[B61] Lee M. R. Scheidweiler K. B. Diao X. X. Akhlaghi F. Cummins A. Huestis M. A. (2018). Oxytocin by intranasal and intravenous routes reaches the cerebrospinal fluid in rhesus macaques: determination using a novel oxytocin assay. *Mol. Psychiatry* 23 115–122. 10.1038/mp.2017.27 28289281 PMC5862033

[B62] Leng G. Ludwig M. (2016). Intranasal oxytocin: myths and delusions. *Biol. Psychiatry* 79 243–250. 10.1016/j.biopsych.2015.05.003 26049207

[B63] Leng G. Sabatier N. (2016). Measuring oxytocin and vasopressin: bioassays, immunoassays and random numbers. *J. Neuroendocrinol.* 28:0.1111/jne.12413. 10.1111/jne.12413 27467712 PMC5096068

[B64] Lewis D. E. O’Reilly M. J. Khuu S. K. Pearson J. (2013). Conditioning the mind’s eye: associative learning with voluntary mental imagery. *Clin. Psychol. Sci.* 1 390–400. 10.1177/2167702613484716

[B65] Li Q. Becker B. Wernicke J. Chen Y. Zhang Y. Li R. (2019). Foot massage evokes oxytocin release and activation of orbitofrontal cortex and superior temporal sulcus. *Psychoneuroendocrinology* 101 193–203. 10.1016/j.psyneuen.2018.11.016 30469087

[B66] Light K. C. Grewen K. M. Amico J. A. (2005). More frequent partner hugs and higher oxytocin levels are linked to lower blood pressure and heart rate in premenopausal women. *Biol. Psychol.* 69 5–21. 10.1016/j.biopsycho.2004.11.002 15740822

[B67] Lindgren L. Westling G. Brulin C. Lehtipalo S. Andersson M. Nyberg L. (2012). Pleasant human touch is represented in pregenual anterior cingulate cortex. *Neuroimage* 59 3427–3432. 22100768 10.1016/j.neuroimage.2011.11.013

[B68] Lund I. Yu L. C. Uvnas-Moberg K. Wang J. Yu C. Kurosawa M. (2002). Repeated massage-like stimulation induces long-term effects on nociception: contribution of oxytocinergic mechanisms. *Eur. J. Neurosci.* 16 330–338. 10.1046/j.1460-9568.2002.02087.x 12169113

[B69] Luo L. Becker B. Geng Y. Zhao Z. Gao S. Zhao W. (2017). Sex-dependent neural effect of oxytocin during subliminal processing of negative emotion faces. *Neuroimage* 162 127–137. 10.1016/j.neuroimage.2017.08.079 28877512

[B70] Ma X. Zhao W. Luo R. Zhou F. Geng Y. Xu L. (2018). Sex-and context-dependent effects of oxytocin on social sharing. *Neuroimage* 183 62–72. 10.1016/j.neuroimage.2018.08.004 30086408

[B71] Martins D. A. Mazibuko N. Zelaya F. Vasilakopoulou S. Loveridge J. Oates A. (2020). Effects of route of administration on oxytocin-induced changes in regional cerebral blood flow in humans. *Nat. Commun.* 11:1160. 10.1038/s41467-020-14845-5 32127545 PMC7054359

[B72] Matthiesen A. S. Ransjö-Arvidson A. B. Nissen E. Uvnäs-Moberg K. (2001). Postpartum maternal oxytocin release by newborns: effects of infant hand massage and sucking. *Birth* 28 13–19. 11264623 10.1046/j.1523-536x.2001.00013.x

[B73] McCullough M. E. Churchland P. S. Mendez A. J. (2013). Problems with measuring peripheral oxytocin: can the data on oxytocin and human behavior be trusted? *Neurosci. Biobehav. Rev.* 37 1485–1492. 10.1016/j.neubiorev.2013.04.018 23665533

[B74] McGlone F. Olausson H. Boyle J. A. Jones-Gotman M. Dancer C. Guest S. (2012). Touching and feeling: differences in pleasant touch processing between glabrous and hairy skin in humans. *Eur. J. Neurosci.* 35 1782–1788. 10.1111/j.1460-9568.2012.08092.x 22594914

[B75] McGlone F. Wessberg J. Olausson H. (2014). Discriminative and affective touch: sensing and feeling. *Neuron* 82 737–755. 10.1016/j.neuron.2014.05.001 24853935

[B76] Mertens G. Krypotos A. M. Engelhard I. M. (2020). A review on mental imagery in fear conditioning research 100 years since the ‘Little Albert’ study. *Behav. Res. Ther.* 126:103556 10.1016/j.brat.2020.103556 32014694

[B77] Minichino A. Cadenhead K. (2017). Mirror neurons in psychiatric disorders: from neuroception to bio-behavioral system dysregulation. *Neuropsychopharmacology* 42:366. 10.1038/npp.2016.220 27909332 PMC5143509

[B78] Mitsui S. Yamamoto M. Nagasawa M. Mogi K. Kikusui T. Ohtani N. (2011). Urinary oxytocin as a noninvasive biomarker of positive emotion in dogs. *Horm. Behav.* 60 239–243. 10.1016/j.yhbeh.2011.05.012 21689655

[B79] Morhenn V. Beavin L. E. Zak P. J. (2012). Massage increases oxytocin and reduces adrenocorticotropin hormone in humans. *Altern. Ther. Health Med.* 18 11–18. 23251939

[B80] Morrison I. (2016). ALE meta-analysis reveals dissociable networks for affective and discriminative aspects of touch. *Hum. Brain. Mapp.* 37 1308–1320. 10.1002/hbm.23103 26873519 PMC5066805

[B81] Nasiri A. Mahmodi M. A. Nobakht Z. (2016). Effect of aromatherapy massage with lavender essential oil on pain in patients with osteoarthritis of the knee: a randomized controlled clinical trial. *Complement Ther. Clin. Pract.* 25 75–80. 10.1016/j.ctcp.2016.08.002 27863613

[B82] Newmark T. S. Bogacki D. F. (2005). The use of relaxation, hypnosis, and imagery in sport psychiatry. *Clin. Sports Med.* 24 973–977. 10.1016/j.csm.2005.06.003 16169457

[B83] Olausson H. Lamarre Y. Backlund H. Morin C. Wallin B. Starck G. (2002). Unmyelinated tactile afferents signal touch and project to insular cortex. *Nat. Neurosci.* 5 900–904. 10.1038/nn896 12145636

[B84] Olausson H. Wessberg J. McGlone F. Vallbo A. (2010). The neurophysiology of unmyelinated tactile afferents. *Neurosci. Biobehav. Rev.* 34 185–191. 10.1016/j.neubiorev.2008.09.011 18952123

[B85] Paloyelis Y. Doyle O. M. Zelaya F. O. Maltezos S. Williams S. C. Fotopoulou A. (2016). A spatiotemporal profile of in vivo cerebral blood flow changes following intranasal oxytocin in humans. *Biol. Psychiatry* 79 693–705. 10.1016/j.biopsych.2014.10.005 25499958

[B86] Parker K. J. Garner J. P. Libove R. A. Hyde S. A. Hornbeak K. B. Carson D. S. (2014). Plasma oxytocin concentrations and OXTR polymorphisms predict social impairments in children with and without autism spectrum disorder. *Proc. Natl. Acad. Sci. U.S.A.* 111 12258–12263. 10.1073/pnas.1402236111 25092315 PMC4143031

[B87] Pawling R. Trotter P. D. McGlone F. P. Walker S. C. (2017). A positive touch: C-tactile afferent targeted skin stimulation carries an appetitive motivational value. *Biol. Psychol.* 129 186–194. 10.1016/j.biopsycho.2017.08.057 28865933

[B88] Perini I. Olausson H. (2015). Seeking pleasant touch: neural correlates of behavioral preferences for skin stroking. *Front. Behav. Neurosci.* 9:8. 10.3389/fnbeh.2015.00008 25698948 PMC4318429

[B89] Perlman A. Fogerite S. G. Glass O. Bechard E. Ali A. Njike V. Y. (2019). Efficacy and safety of massage for osteoarthritis of the knee: a randomized clinical trial. *J. Gen. Intern. Med.* 34 379–386. 10.1007/s11606-018-4763-5 30543021 PMC6420526

[B90] Quintana D. S. Rokicki J. van der Meer D. Alnaes D. Kaufmann T. Cóordova-Polomera A. (2018). Oxytocin pathway gene networks in the human brain. *Nat. Commun.* 10:668. 10.1038/s41467-019-08503-8 30737392 PMC6368605

[B91] Quintana D. S. Westlye L. T. Alnæs D. Rustan ØG. Kaufmann T. Smerud K. (2016). Low dose intranasal oxytocin delivered with Breath Powered device dampens amygdala response to emotional stimuli: a peripheral effect-controlled within-subjects randomized dose-response fMRI trial. *Psychoneuroendocrinology* 69 180–188. 10.1016/j.psyneuen.2016.04.010 27107209

[B92] Quintana D. S. Lischke A. Grace S. Scheele D. Ma Y. Becker B. (2020). Advances in the field of intranasal oxytocin research: lessons learned and future directions for clinical research. *Mol. Psychiatry* 1–12. 10.1038/s41380-020-00864-7 32807845 PMC7815514

[B93] Riem M. M. Bakermans-Kranenburg M. J. Pieper S. Tops M. Boksem M. A. Vermeiren R. R. (2011). Oxytocin modulates amygdala, insula, and inferior frontal gyrus responses to infant crying: a randomized controlled trial. *Biol. Psychiatry* 70 291–297. 10.1016/j.biopsych.2011.02.006 21470595

[B94] Rizzolatti G. Fabbri-Destro M. (2008). The mirror system and its role in social cognition. *Curr. Opin. Neurobiol.* 18 179–184. 10.1016/j.conb.2008.08.001 18706501

[B95] Rolls E. T. (2010). The affective and cognitive processing of touch, oral texture, and temperature in the brain. *Neurosci. Biobehav. Rev.* 34 237–245. 10.1016/j.neubiorev.2008.03.010 18468687

[B96] Rolls E. T. O’Doherty J. Kringelbach M. L. Francis S. Bowtell R. McGlone F. (2003). Representations of pleasant and painful touch in the human orbitofrontal and cingulate cortices. *Cereb. Cortex.* 13 308–317. 10.1093/cercor/13.3.308 12571120

[B97] Sailer U. Triscoli C. Häggblad G. Hamilton P. Olausson H. Croy I. (2016). Temporal dynamics of brain activation during 40 minutes of pleasant touch. *NeuroImage* 139 360–367. 10.1016/j.neuroimage.2016.06.031 27338514

[B98] Scheele D. Kendrick K. M. Khouri C. Kretzer E. Schläpfer T. E. Stoffel-Wagner B. (2014a). An oxytocin-induced facilitation of neural and emotional responses to social touch correlates inversely with autism traits. *Neuropsychopharmacology* 39 2078–2085. 10.1038/npp.2014.78 24694924 PMC4104346

[B99] Scheele D. Striepens N. Kendrick K. M. Schwering C. Noelle J. Wille A. (2014b). Opposing effects of oxytocin on moral judgment in males and females. *Hum. Brain. Mapp.* 35 6067–6076. 10.1002/hbm.22605 25094043 PMC6868938

[B100] Scheele D. Wille A. Kendrick K. M. Stoffel-Wagner B. Becker B. Güntürkün O. (2013). Oxytocin enhances brain reward system responses in men viewing the face of their female partner. *Proc. Natl. Acad. Sci. U.S.A.* 110 20308–20313. 10.1073/pnas.1314190110 24277856 PMC3864312

[B101] Schneiderman I. Zagoory-Sharon O. Leckman J. F. Feldman R. (2012). Oxytocin during the initial stages of romantic attachment: relations to couples’ interactive reciprocity. *Psychoneuroendocrinology* 37 1277–1285. 10.1016/j.psyneuen.2011.12.021 22281209 PMC3936960

[B102] Schoen S. A. Miller L. J. Green K. E. (2008). Pilot study of the sensory over-responsivity scales: assessment and inventory. *Am. J. Occup. Ther.* 62 393–406. 10.5014/ajot.62.4.393 18712002

[B103] Sindermann C. Luo R. Becker B. Kendrick K. M. Montag C. (2020). The role of oxytocin on self-serving lying. *Brain Behav.* 10:e01518. 10.1002/brb3.1518 31930678 PMC7010580

[B104] Sliz D. Smith A. Wiebking C. Northoff G. Hayley S. (2012). Neural correlates of a single-session massage treatment. *Brain Imaging Behav.* 6 77–87. 10.1007/s11682-011-9146-z 22261925 PMC3282900

[B105] Spengler F. B. Schultz J. Scheele D. Essel M. Maier W. Heinrichs M. (2017). Kinetics and dose dependency of intranasal oxytocin effects on amygdala reactivity. *Biol. Psychiatry* 82 885–894. 10.1016/j.biopsych.2017.04.015 28629540

[B106] Spielberger C. D. Gorsuch R. L. Lushene R. Vagg P. R. Jacobs G. A. (1983). *Manual for the State-Trait Anxiety Inventory.* Palo Alto, CA: Consulting Psychologists Press.

[B107] Stock S. Uvnäs-Moberg K. (1988). Increased plasma levels of oxytocin in response to afferent electrical stimulation of the sciatic and vagal nerves and in response to touch and pinch in anaesthetized rats. *Acta Physiol. Scand.* 132 29–34. 10.1111/j.1748-1716.1988.tb08294.x 3223304

[B108] Striepens N. Kendrick K. M. Hanking V. Landgraf R. Wüllner U. Maier W. (2013). Elevated cerebrospinal fluid and blood concentrations of oxytocin following its intranasal administration in humans. *Sci. Rep.* 3:3440. 10.1038/srep03440 24310737 PMC3853684

[B109] Striepens N. Scheele D. Kendrick K. M. Becker B. Schäfer L. Schwalba K. (2012). Oxytocin facilitates protective responses to aversive social stimuli in males. *Proc. Natl. Acad. Sci. U.S.A.* 109 18144–18149. 10.1073/pnas.1208852109 23074247 PMC3497762

[B110] Szeto A. McCabe P. M. Nation D. A. Tabak B. A. Rossetti M. A. McCullough M. E. (2011). Evaluation of enzyme immunoassay and radioimmunoassay methods for the measurement of plasma oxytocin. *Psychosom. Med.* 73 393–400. 10.1097/PSY.0b013e31821df0c2 21636661 PMC3118424

[B111] Takahashi T. Babygirija R. R. Ludwig K. (2015). Anti-stress effect of hypothalamic oxytocin-Importance of somatosensory stimulation and social buffering. *Int. J. Neurol. Res.* 1 96–101. 10.17554/j.issn.2313-5611.2015.01.18

[B112] Tanaka A. Furubayashi T. Arai M. Inoue D. Kimura S. Kiriyama A. (2018). Delivery of oxytocin to the brain for the treatment of autism spectrum disorder by nasal application. *Mol. Pharm.* 15 1105–1111. 10.1021/acs.molpharmaceut.7b00991 29338251

[B113] Taurines R. Schwenck C. Lyttwin B. Schecklmann M. Jans T. Reefschläger L. (2014). Oxytocin plasma concentrations in children and adolescents with autism spectrum disorder: correlation with autistic symptomatology. *Atten. Defic. Hyperact. Disord.* 6 231–239. 10.1007/s12402-014-0145-y 24989441

[B114] Torem M. S. (1992). Therapeutic imagery enhanced by hypnosis. *Psychiatr. Med.* 10 1–12. 1283921

[B115] Torrubia R. Avila C. Moltó J. Caseras X. (2001). The Sensitivity to Punishment and Sensitivity to Reward Questionnaire (SPSRQ) as a measure of Gray’s anxiety and impulsivity dimensions. *Pers. Indiv. Differ.* 31 837–862. 10.1016/S0191-8869(00)00183-5

[B116] Tsuji S. Yuhi T. Furuhara K. Ohta S. Shimizu Y. Higashida H. (2015). Salivary oxytocin concentrations in seven boys with autism spectrum disorder received massage from their mothers: a pilot study. *Front. Psychiatry* 6:58. 10.3389/fpsyt.2015.00058 25954210 PMC4404976

[B117] Uddin L. Q. (2015). Salience processing and insular cortical function and dysfunction. *Nat. Rev. Neurosci.* 16 55–61. 10.1038/nrn3857 25406711

[B118] Uddin L. Q. Nomi J. S. Hébert-Seropian B. Ghaziri J. Boucher O. (2017). Structure and function of the human insula. *J. Clin. Neurophysiol.* 34 300–306. 10.1097/WNP.0000000000000377 28644199 PMC6032992

[B119] Uvnäs-Moberg K. Handlin L. Kendall-Tackett K. Petersson M. (2019). Oxytocin is a principal hormone that exerts part of its effects by active fragments. *Med. Hypotheses* 133:109394. 10.1016/j.mehy.2019.109394 31525634

[B120] Uvnäs-Moberg K. Handlin L. Petersson M. (2015). Self-soothing behaviors with particular reference to oxytocin release induced by non-noxious sensory stimulation. *Front. Psychol.* 5:1529. 10.3389/fpsyg.2014.01529 25628581 PMC4290532

[B121] Uvnäs-Moberg K. Handlin L. Petersson M. (2020). Neuroendocrine mechanisms involved in the physiological effects caused by skin-to-skin contact–With a particular focus on the oxytocinergic system. *Infant. Behav. Dev.* 61:101482 10.1016/j.infbeh.2020.101482 32919112

[B122] Uvnäs-Moberg K. Petersson M. (2010). “Role of oxytocin and oxytocin related effects in manual therapies,” in *The Science and Clinical Application of Manual Therapy*, eds King H. H. Jänig W. Patterson M. M. (Amsterdam: Elsevier), 147–162.

[B123] Vagnoli L. Bettini A. Amore E. De Masi S. Messeri A. (2019). Relaxation-guided imagery reduces perioperative anxiety and pain in children: a randomized study. *Eur. J. Pediatr.* 178 913–921. 10.1007/s00431-019-03376-x 30944985

[B124] Velandia M. (2012). *Parent-Infant Skin-to-Skin Contact Studies: Parent-Infant Interaction and Oxytocin Levels During Skin-to-Skin Contact After Cesarean Section and Motherinfant Skin-to-Skin Contact as Treatment for Breastfeeding Problems.* PhD Thesis, Karolinska Institutet, Sweden.

[B125] Vittner D. McGrath J. Robinson J. Lawhon G. Cusson R. Eisenfeld L. (2018). Increase in oxytocin from skin-to-skin contact enhances development of parent–infant relationship. *Biol. Res. Nurs.* 20 54–62. 10.1177/1099800417735633 29017336

[B126] Voos A. C. Pelphrey K. A. Kaiser M. D. (2013). Autistic traits are associated with diminished neural response to affective touch. *Soc. Cogn. Affect. Neurosci.* 8 378–386. 10.1093/scan/nss009 22267520 PMC3624948

[B127] Walker S. C. Trotter P. D. Swaney W. T. Marshall A. McGlone F. P. (2017). C-tactile afferents: cutaneous mediators of oxytocin release during affiliative tactile interactions? *Neuropeptides* 64 27–38. 10.1016/j.npep.2017.01.001 28162847

[B128] Watson D. Clark L. A. Tellegen A. (1988). Development and validation of brief measures of positive and negative affect: the PANAS scales. *J. Pers. Soc. Psychol.* 54 1063–1070. 10.1037//0022-3514.54.6.1063 3397865

[B129] Wilhelm F. H. Kochar A. S. Roth W. T. Gross J. J. (2001). Social anxiety and response to touch: incongruence between self-evaluative and physiological reactions. *Biol. Psychol.* 58 181–202. 10.1016/s0301-0511(01)00113-2 11698114

[B130] Xu X. Liu C. Zhou X. Chen Y. Gao Z. Zhou F. (2019). Oxytocin facilitates self-serving rather than altruistic tendencies in competitive social interactions via orbitofrontal cortex. *Int. J. Neuropsychopharmacol.* 22 501–512. 10.1093/ijnp/pyz028 31152588 PMC6672625

[B131] Yamamoto Y. Higashida H. (2020). RAGE regulates oxytocin transport into the brain. *Commun. Biol.* 3:70. 10.1038/s42003-020-0799-2 32054984 PMC7018824

[B132] Yao S. Becker B. Zhao W. Zhao Z. Kou J. Ma X. (2018a). Oxytocin modulates attention switching between interoceptive signals and external social cues. *Neuropsychopharmacology* 43 294–301. 10.1038/npp.2017.189 28836577 PMC5729568

[B133] Yao S. Zhao W. Geng Y. Chen Y. Zhao Z. Ma X. (2018b). Oxytocin facilitates approach behavior to positive social stimuli via decreasing anterior insula activity. *Int. J. Neuropsychopharmacol.* 21 918–925. 10.1093/ijnp/pyy068 30085122 PMC6165955

[B134] Zhang H.-F. Dai Y.-C. Wu J. Jia M.-X. Zhang J.-S. Shou X.-J. (2016). Plasma oxytocin and arginine-vasopressin levels in children with autism spectrum disorder in China: associations with symptoms. *Neurosci. Bull.* 32 423–432. 10.1007/s12264-016-0046-5 27342432 PMC5563759

[B135] Zhao W. Geng Y. Luo L. Zhao Z. Ma X. Xu L. (2017). Oxytocin increases the perceived value of both self-and other-owned items and alters medial prefrontal cortex activity in an endowment task. *Front. Hum. Neurosci.* 11:272. 10.3389/fnhum.2017.00272 28588469 PMC5440465

[B136] Zhao W. Yao S. Li Q. Geng Y. Ma X. Luo L. (2016). Oxytocin blurs the self-other distinction during trait judgments and reduces medial prefrontal cortex responses. *Hum. Brain Mapp.* 37 2512–2527. 10.1002/hbm.23190 27016006 PMC6867482

