# Supplementary material for: The Effects of Intranasal Oxytocin on Neural and Behavioral Responses to Social Touch in the Form of Massage
Source: Front Neurosci. 2020 Dec 4;14:589878. doi: 10.3389/fnins.2020.589878 (PMC7746800; doi:10.3389/fnins.2020.589878)
Supplement: Supplementary file 1 [file Table_1.DOCX]

Supplementary Material

# Results

To explore whether intranasal treatment had effects on personal mood, we analyzed individual self-reported mood scores measured by the PANAS. A two way ANOVA analysis on PANAS scores was performed with treatment (OXT vs. PLC) and measured time (T1: pre-treatment vs. T2: 35 min post-treatment vs. T3: post-manual massage vs. T4: post-machine massage) as within-subject factors. For the positive mood scores, there was a significant main effect of time-point (F(3, 135) = 10.405, p < 0.001, ƞ^2^ = 0.188) due to ratings being higher post massage (T3 and T4) compared to pre-treatment (T1) and post-treatment (T2) and with no difference between T1 and T2. There was no significant main effect of treatment (p = 0.17) or treatment × time point interaction (p = 0.22). An exploratory t-test analysis with Bonferonni correction revealed that only after the manual massage, when the subjects were treated with OXT they gave higher positive mood scores compared with under PLC (p = 0.015). There were no other significant differences in PANAS rating scores for other time points between OXT and PLC. Subjects rated higher positive mood after the manual massage compared with post-treatment (p < 0.01) for both treatment sessions. Following OXT treatment subjects gave increased scores after the manual massage compared with pre-treatment (p = 0.034) and post-machine massage (p = 0.006), but not under PLC (ps > 0.24). For negative PANAS mood scores, results showed that there were no significant main effects and interactions (ps > 0.10).

# Supplementary Figures and Tables

## Supplemental tables

**Table S1.** Descriptive statistics of questionnaire scores (mean ± sd)

| Measurements | Range | Mean ± sd |
| --- | --- | --- |
| Beck Depression Inventory II | 0 - 25 | 6.44 ± 6.74 |
| State-Trait Anxiety Inventory |  |  |
| - State anxiety | 25 - 55 | 36.96 ± 7.97 |
| - Trait anxiety | 27 - 61 | 40.28 ± 7.42 |
| Liebowitz Social Anxiety Scale |  |  |
| - Fear | 3 - 48 | 20.84 ± 11.25 |
| - Avoid | 6 - 43 | 18.96 ± 10.45 |
| Empathy Quotient | 20 - 69 | 38.82 ± 10.26 |
| Sensitivity to Punishment and Sensitivity to Reward Questionnaire | | |
| - Sensitivity to Punishment | 1 - 23 | 11.22 ± 6.00 |
| - Sensitivity to Reward | 7 - 22 | 14.98 ± 3.84 |
| Autism Spectrum Quotient | 10 - 33 | 19.91 ± 5.46 |
| Social Touch Questionnaire | 18 - 60 | 40.78 ± 7.49 |
| Sensory-Over Responsivity Scales | 4 - 57 | 21.49 ± 1.74 |
